# The prognostic value of blood lactate levels relative to that of vital signs in the pre-hospital setting: a pilot study

**DOI:** 10.1186/cc7159

**Published:** 2008-12-17

**Authors:** Tim C Jansen, Jasper van Bommel, Paul G Mulder, Johannes H Rommes, Selma JM Schieveld, Jan Bakker

**Affiliations:** 1 Department of Intensive Care, Erasmus MC University Medical Center, PO Box 2040, 3000 CA, Rotterdam, The Netherlands; 2 Department of Epidemiology & Biostatistics, Erasmus MC University Medical, PO Box 2040, 3000 CA, Rotterdam, The Netherlands; 3 Department of Intensive Care, Gelre Hospital, location Lukas, PO Box 9014, 7300 DS Apeldoorn, The Netherlands

## Abstract

**Introduction:**

A limitation of pre-hospital monitoring is that vital signs often do not change until a patient is in a critical stage. Blood lactate levels are suggested as a more sensitive parameter to evaluate a patient's condition. The aim of this pilot study was to find presumptive evidence for a relation between pre-hospital lactate levels and in-hospital mortality, corrected for vital sign abnormalities.

**Methods:**

In this prospective observational study (n = 124), patients who required urgent ambulance dispatching and had a systolic blood pressure below 100 mmHg, a respiratory rate less than 10 or more than 29 breaths/minute, or a Glasgow Coma Scale (GCS) below 14 were enrolled. Nurses from Emergency Medical Services measured capillary or venous lactate levels using a hand-held device on arrival at the scene (T1) and just before or on arrival at the emergency department (T2). The primary outcome measured was in-hospital mortality.

**Results:**

The average (standard deviation) time from T1 to T2 was 27 (10) minutes. Non-survivors (n = 32, 26%) had significantly higher lactate levels than survivors at T1 (5.3 vs 3.7 mmol/L) and at T2 (5.4 vs 3.2 mmol/L). Mortality was significantly higher in patients with lactate levels of 3.5 mmol/L or higher compared with those with lactate levels below 3.5 mmol/L (T1: 41 vs 12% and T2: 47 vs 15%). Also in the absence of hypotension, mortality was higher in those with higher lactate levels. In a multivariable Cox proportional hazard analysis including systolic blood pressure, heart rate, GCS (all at T1) and delta lactate level (from T1 to T2), only delta lactate level (hazard ratio (HR) = 0.20, 95% confidence interval (CI) = 0.05 to 0.76, p = 0.018) and GCS (HR = 0.93, 95% CI = 0.88 to 0.99, p = 0.022) were significant independent predictors of in-hospital mortality.

**Conclusions:**

In a cohort of patients that required urgent ambulance dispatching, pre-hospital blood lactate levels were associated with in-hospital mortality and provided prognostic information superior to that provided by the patient's vital signs. There is potential for early detection of occult shock and pre-hospital resuscitation guided by lactate measurement. However, external validation is required before widespread implementation of lactate measurement in the out-of-hospital setting.

## Introduction

An important limitation of patient monitoring in the pre-hospital phase is that the standard vital signs such as heart rate and blood pressure often do not change until a patient reaches a critical stage [1-3]. Pain and anxiety, contributing to increased sympathetic tone, influence these vital signs and render them insensitive for monitoring the adequacy of tissue perfusion [4]. Many patients who appear to be haemodynamically stable based on normal vital signs have increased blood lactate levels ('occult hypoperfusion' or 'compensated shock') [1,5]; as a result, lactate levels are often considered to be better resuscitation endpoints than standard vital signs [6].

Lactate levels are commonly used to stratify risk and to assess adequacy of resuscitation in the intensive care unit (ICU) [7,8] and in the emergency department (ED) [9-11], but are not currently used in the pre-hospital setting [12]. As it is possible to measure blood lactate levels on-site using a fast and accurate hand-held analyser on capillary or venous blood [13,14], lactate monitoring can be transferred from the hospital to the pre-hospital setting. The aim of this pilot study was to find presumptive evidence for a relation between pre-hospital lactate levels and patient outcome. We hypothesised that pre-hospital blood lactate measurements would enable the prediction of in-hospital mortality and that this prognostic value would be independent of commonly available standard vital parameters.

## Materials and methods

### Study design

This was a prospective observational cohort study.

### Setting

A Dutch Emergency Medical Service (EMS), referring to three university-affiliated hospitals, dispatched ambulances that were staffed by certified EMS nurses with two years of postgraduate training in a critical care setting (ICU, cardiac care unit, anaesthesiology or ED) and one year of training in EMS-specific procedures.

### Selection of participants

A convenience sample of patients were enrolled who required urgent ambulance dispatching and had a systolic blood pressure below 100 mmHg, respiratory rate of less than 10 or more than 29 breaths/minute or a Glasgow Coma Scale (GCS) of less than 14 on arrival of the ambulance. Exclusion criteria were the unavailability of a first lactate measurement or epileptic seizures, in which case hyperlactataemia is prognostically less sensitive [15]. The study was approved by the Medical Ethics Committee, which waived the need for obtaining informed consent.

### Interventions

Pre-hospital treatment was provided by EMS nurses according to Dutch national ambulance protocols (Landelijk Protocol Ambulancezorg (LPA)). These protocols are in accordance with the pre-hospital and advanced trauma life support guidelines of the National Association of Emergency Medical Technicians, based on the Advanced Trauma Life Support standard of the American College of Surgeons. During the study period from June 1997 to November 1998, LPA version 4 (1996 to 1999) was used. When compared with the current version 7 (2007 to 2010), most protocols were similar.

### Methods of measurements and data collection

The first lactate measurement (T1) was performed by EMS nurses as soon as possible after arrival at the scene (before any pre-hospital treatment); the second measurement (T2) was obtained just before or on arrival at the ED (after pre-hospital treatment). The lactate level was measured in venous or capillary blood immediately after blood was drawn (at T1 or T2) using a point-of-care hand-held lactate analyser (Accutrend, Roche Diagnostics, Mannheim, Germany). This is a small, battery-powered, reflectance photometer with a turnaround time of 60 seconds that uses chemistry test strips on which a drop of blood is applied. Hospital physicians were not informed about the lactate levels collected by the EMS nurses. Other obtained data at both T1 and T2 included heart rate, diastolic and systolic blood pressures, peripheral oxygen saturation obtained by pulse oxymeter (SpO_2_) and GCS. SpO_2 _was regarded as a binary variable, which was defined as abnormal if it was lower than 92% or if the pulse oxymeter signal could not be retrieved because of inadequate peripheral perfusion (n = 25). If heart rate and blood pressure readings could not be obtained because of cardiac arrest at T1 (asystole or ventricular fibrillation, n = 11), we considered these values as 0 (this was only done at T1, not at T2).

### Outcome measures

The primary outcome measured was in-hospital mortality.

### Primary data analysis

Because lactate levels were not normally distributed, they were logarithmically transformed before analysis. To evaluate the prognostic accuracy of the lactate levels, receiver operating characteristic (ROC) curves for in-hospital mortality were constructed and area under the ROC curves (AUROC) were calculated. Using ROC-curve analysis, we defined appropriate cut-off values (which are not available for the pre-hospital setting) and calculated sensitivity, specificity, positive predictive values (PPV) and negative predictive values (NPV). In order to identify patients who were likely to die, the test had to be sensitive while remaining specific [16] and had to have an acceptable PPV [17]. Mortality rates of patients with high or low lactate levels were compared using a chi squared test or Fisher's exact test if necessary, based on sample size.

In order to identify independent predictors of in-hospital death, adjusted for standard variables available in the pre-hospital setting, a multivariable Cox proportional hazards (PH) model was constructed. The variables systolic blood pressure, heart rate, GCS and the change in lactate level from T1 to T2 were simultaneously entered in this model (the number of variables was restricted to four to reduce the possibility of overfitting). A backward elimination method was used, in which each step removed the variable with the highest p-value above 0.10 according to the likelihood ratio test. Interaction between all variables was not tested because of the risk of overfitting. The PH assumption was confirmed by entering variable-by-time interaction terms one by one, with time on the log scale. By choosing Cox PH instead of logistic regression analysis, we took account of the time of death, rather than just dead (yes or no) in the analysis. Statistical analyses were performed using SPSS version 11.0.1/12.0.1 (SPSS, Inc., Chicago, IL, USA).

## Results

### Characteristics of study subjects

We enrolled 135 patients. Three patients were excluded because of a missing first lactate measurement and eight patients had epileptic seizures. The baseline characteristics of the remaining 124 patients are described in Table 1. The mean (standard deviation) time at the scene from arrival to departure of the ambulance was 16 (8) minutes. Mean duration of the subsequent transfer to the ED was 11 (6) minutes. The total time from arrival at the scene to arrival in the ED was 27 (10) minutes.

**Table 1:** Baseline characteristics

	**Total:** **n = 124**	**Non-survivors:** **n = 32**	**Survivors:** **n = 92**
Age (years, ± SD)	62 ± 19	68 ± 14 *	59 ± 20 *
Sex (n, % male)	73 (59%)	22 (69%)	51 (55%)
Intensive care unit admission (n, %)	57 (46%)	15 (47%)	42 (46%)
Length of stay in hospital (days, ± SD)	13 ± 21	3 ± 6 *	17 ± 23 *
Time arrival ambulance to ED (minutes, ± SD)	27 ± 9	29 ± 10	26 ± 10
Ambulance diagnosis (n, %):			
- cardiac arrest	12 (10%)	8 (25%) *	4 (4%) *
- myocardial infarction	17 (14%)	2 (6%)	15 (16%)
- other cardiological disorders	8 (6%)	1 (3%)	7 (8%)
- sepsis	8 (6%)	4 (13%)	4 (4%)
- haemorrhage	10 (8%)	3 (9%)	7 (8%)
- neurological disorder	19 (15%)	9 (28%) *	10 (11%) *
- trauma without severe traumatic brain injury	18 (15%)	2 (6%)	16 (17%)
- trauma with severe traumatic brain injury	2 (2%)	1 (3%)	1 (1%)
- attempted suicide	4 (3%)	0 (0%)	4 (4%)
- others	26 (21%)	2 (6%) *	24 (26%) *

Continuous data are presented as mean ± standard deviation (SD). Binary data are presented as n (percentage of total, non-survivors or survivors). * p < 0.05. ED = emergency department.

### Before pre-hospital treatment (T1)

**Figure 1:**
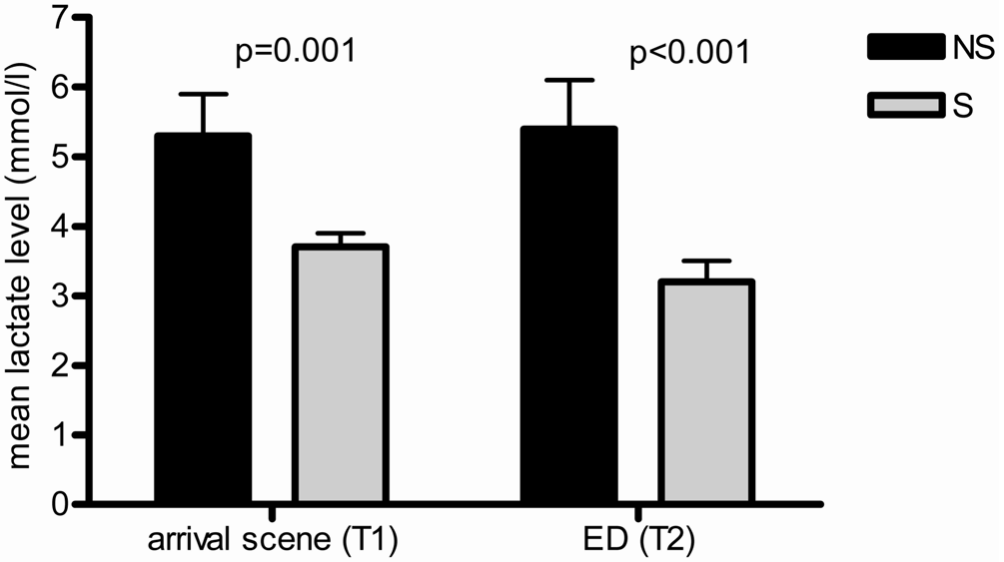
**Mean lactate levels in survivors (S) and non-survivors (NS) on arrival of the ambulance at the scene (T1) and just before or on arrival at the emergency department (T2)**. Arrow bar represents standard error. Number of patients at T1: n = 124 and at T2: n = 106.

Of the 124 patients who were included in the study on arrival of the ambulance at the scene, 92 survived and 32 died. Compared with the survivors, the non-survivors had a lower systolic blood pressure, lower GCS, more often an abnormal SpO_2 _and an older age (Table 2). Heart rates were not significantly different. Lactate levels were higher in the non-survivors (Figure 1).

**Table 2:** Vital signs in survivors (S) and non-survivors (NS) on arrival of the ambulance on the scene (T1) and just before or on arrival at the emergency department (T2)

	**T1**		**T2**	
		
	**NS**	**S**	**NS**	**S**
Heart rate (beats/minute, ± SD)	75 ± 51	89 ± 30	90 ± 40	90 ± 22
Systolic arterial pressure (mmHg, ± SD)	101 ± 66 *	126 ± 41 *	132 ± 43	136 ± 28
Mean arterial pressure (mmHg, ± SD)	86 ± 56 *	108 ± 35 *	113 ± 36	117 ± 23
SpO_2 _< 92% or no signal (n, %)	23 (72%) *	34 (37%) *	12 (43%)	22 (28%)
GCS (± SD)	8 ± 6 *	13 ± 4 *	9 ± 6 *	13 ± 4 *

Continuous data are presented as mean ± standard deviation (SD). Binary data are presented as n (percentage non-survivors or survivors). Number of patients: T1 n = 124 (32 NS and 92 S), T2 n = 106 (28 NS and 88 S). * p < 0.05. SpO_2 _= peripheral oxygen saturation, GCS= Glasgow Coma Scale.

**Figure 2:**
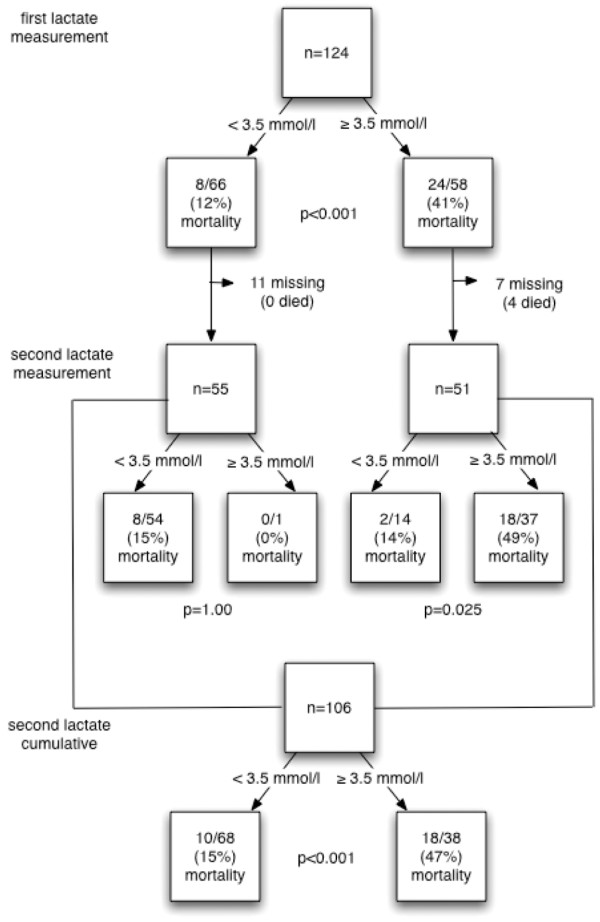
**Patient survival according to lactate levels below or above the cut-off threshold of 3.5 mmol/L**.

At T1, the AUROC of lactate for in-hospital death was 0.69 (95% confidence interval (CI) = 0.58 to 0.80, p = 0.001). We established a lactate level of 3.5 mmol/L as the best cut-off point for T1. A lactate level of 3.5 mmol/L or more was 75% sensitive (95% CI = 60 to 90%) and 63% specific (95% CI = 53 to 73%) for prediction of death, with a PPV of 41% (95% CI = 29 to 54%) and a NPV of 88% (95% CI = 80 to 96%). Mortality in patients with a high lactate level was 41% (95% CI = 29 to 54%), compared with 12% (95% CI = 4 to 20%) for those with a lower level (Figure 2). Patients with high lactate levels also had lower systolic blood pressures (100 vs 137 mmHg, p < 0.001), lower GCS (10 vs 14, p < 0.001), more often an abnormal SpO_2 _(74 vs 21%, p < 0.001) and were more often admitted to the ICU (57 vs 36%, p = 0.022).

**Figure 3:**
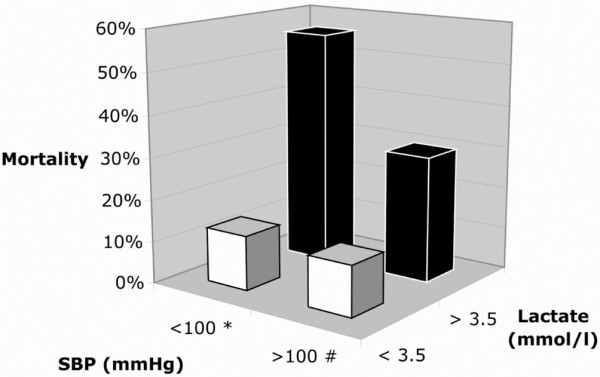
**In-hospital mortality stratified by systolic blood pressure and blood lactate level measured at arrival of the ambulance at the scene (T1)**. *p = 0.046 ^#^p = 0.032 Number of patients per group: low systolic blood pressure (SBP)/low lactate n = 8, low SBP/high lactate n = 25, high SBP/low lactate n = 58, high SBP/high lactate n = 33.

At T1, 33 patients had a systolic blood pressure below 100 mmHg. To adjust for the presence of a systolic blood pressure below 100 mmHg [18], a stratified analysis was performed, which showed that lactate was still significantly associated with mortality (Figure 3).

### After pre-hospital treatment (T2)

Follow-up lactate measurements were available for 106 patients. Of these patients, 78 survived and 28 died in the hospital. Compared with the survivors, the non-survivors had a lower GCS (9 vs 13, p < 0.001) and a higher lactate level (Figure 2). Systolic blood pressure, heart rate and SpO_2 _did not significantly differ between the two groups.

At T2, the AUROC was 0.72 (95% CI = 0.60 to 0.84, p = 0.001). Here, 3.5 mmol/L was again considered as the most appropriate cut-off point with a sensitivity for death of 64% (95% CI = 47 to 82%), specificity of 74% (95% CI = 65 to 84%), PPV of 47% (95% CI = 31 to 63%) and a NPV of 85% (95% CI = 77 to 94%). In the high lactate group, 47% (95% CI = 31 to 63%) of the patients died, while only 15% (95% CI = 6 to 23%) of those with a lower lactate level died (Figure 2). Additionally, patients in the high lactate group had a lower systolic blood pressure (125 vs 140 mmHg, p = 0.017), lower GCS (10 vs 13, p = 0.002), more often an abnormal SpO_2 _(50 vs 22%, p = 0.003) and were more often admitted to the ICU (71 vs 35%, p < 0.001).

Eleven patients had a systolic blood pressure below 100 mmHg at T2. In the other patients with a systolic blood pressure of 100 mmHg or above (n = 95), mortality rates remained significantly higher in those with high (47%, 14 out of 30) versus low lactate levels (15%, 10 out of 65, p = 0.001).

When examining the evolution of lactate during the pre-hospital phase, the lactate level, on average, increased 0.1 mmol/L in non-survivors, whereas in survivors it decreased 0.6 mmol/L (p = 0.044). This evolution of lactate from T1 to T2 had prognostic significance even after the effect of the other parameters (systolic blood pressure, heart rate and GCS) had been taken into account in the multivariable Cox PH model. Of the variables, only the change in lactate level and the GCS were independently associated with in-hospital mortality (Table 3). The hazard of death decreased by 80% (95% CI = 24 to 95%) for every 63% decrease of the lactate level at T2 relative to the level at T1 (i.e. a larger decrease in lactate during pre-hospital treatment was associated with decreased mortality). Although a model with six instead of four entered variables is a possible overfit, adding age and SpO_2 _to the start model resulted in a final model in which delta lactate remained independently associated with in-hospital mortality (with equal hazard ratio, 95% CI and p value, data not shown).

**Table 3:** Multivariable Cox proportional hazards model for the identification of independent variables associated with in-hospital death

Variable	Start model			Final model		
		
	HR	95% CI	p value	HR	95% CI	p value
Δ ln(lactate) T1 to T2*	0.20	0.05 to 0.79	0.022	0.20	0.05 to 0.76	0.018
SBP T1 _per mmHg_	1.00	0.99 to 1.01	0.56	Not in model		0.87
Heart rate T1 _per beat/minute_	1.01	0.99 to 1.02	0.47	Not in model		0.66
GCS T1 _per unit_	0.93	0.87 to 0.99	0.034	0.93	0.88 to 0.99	0.022

The variables were simultaneously entered in the model (start model). A backward elimination method was used to construct the final model.

* Δ ln(lactate) T1 to T2: for every 63% decrease (100*(1-(1/e)) = 63%) of the lactate level at T2 relative to the level at T1, the hazard of death decreased by 80% (100 (1-HR)) in the final model (95% CI = 24 to 95%). e = 2.71828, GCS = Glasgow Coma Scale, HR = hazard ratio, ln = natural logarithm, SBP = systolic blood pressure, T1 = on arrival of the ambulance on the scene, T2 = just before or on arrival at the emergency department.

### Subgroup of patients without cardiac arrest

To test the hypothesis that blood lactate levels remained predictive for outcome in a population that is not obviously in circulatory shock, we repeated the analyses in the subgroup of patients without cardiac arrest. In addition, this would correct for possible negation of the association of tachycardia with mortality because of the coding of heart rate as 0 in cases of cardiac arrest.

Twelve patients had cardiac arrest at T1. Four patients died out-of-hospital (before T2). Of the eight patients with return of spontaneous circulation at T2, four died during hospital admission and four survived. In the subgroup excluding the 12 patients with cardiac arrest (n = 112, in-hospital mortality 21%), lactate level remained a prognostic marker for in-hospital death. The AUROC was 0.66 (95% CI = 0.52 to 0.80, p = 0.015) at T1 and 0.69 (95% CI = 0.55 to 0.82, p = 0.007) at T2. A lactate level of 3.5 mmol/L remained the most appropriate cut-off value at both time points. Using this value at T1, mortality was 35% (95% CI = 21 to 49%) in the group with high lactate levels compared with 12% (95% CI = 4 to 20%) in the group with low lactate levels (p = 0.005). At T2, this was 43% (95% CI = 26 to 61%) compared with 15% (95% CI = 11 to 19%)(p = 0.002). In the final model of multivariable Cox PH analysis performed in the non-cardiac arrest patients, the effect of the change in lactate levels from T1 to T2 remained equally strong with a hazard ratio of 0.22 (95% CI = 0.04 to 1.11), but it was not statistically significant (p = 0.067).

## Discussion

Our results show that in a cohort of patients that required urgent ambulance dispatching, pre-hospital blood lactate levels were associated with in-hospital mortality. In addition, lactate was more sensitive in identifying patients at risk of death than the conventional vital parameters such as systolic blood pressure and heart rate.

The mortality rate of 41% for patients with a first lactate level of 3.5 mmol/L or more indicates that a high-risk population could be identified immediately on arrival of the ambulance at the scene. This was clinically relevant because a simple procedure such as measurement of lactate levels increased the ability to predict death from 26% (pre-test probability or study population mortality) to 41% at T1 and 47% at T2 (post-test probability or PPV). Furthermore, the NPV of 88% demonstrated that low lactate levels identified patients with a low risk of dying. Our study found that a cut-off value of 3.5 mmol/L for the out-of-hospital setting is close to 4.0 mmol/L, which was found to have prognostic significance in the ED [7,9,19]. The prognostic accuracy of pre-hospital lactate levels for predicting in-hospital death, as expressed by AUROC, sensitivity and specificity, was comparable with values found in the ED and ICU setting [5,7,9,19]. Aside from the prognostic information obtained from single lactate measurements, our data also emphasised the value of serial measurements in which the response to administered pre-hospital therapy could be monitored [10,20].

Importantly, the prognostic value of lactate was independent of vital signs. In particular, the association between hyperlactataemia and mortality was not confounded by simultaneous hypotension. Our observation that lactate was a more sensitive marker is in line with earlier studies in the ED or ICU describing the phenomenon of occult hypoperfusion [1,5,11,20-23]. Apparently, compensated shock in which there are signs of tissue hypoperfusion despite the presence of stable vital signs is equally important in the pre-hospital setting. Insufficient oxygen delivery might have been an important cause of hyperlactataemia in our patients, particularly in the earliest phase of disease presentation as was the case in our study [24-26]. In addition, increased aerobic metabolism [27] and reduced clearance [28] might have also contributed to the increased blood lactate levels early in critical illness when blood pressure and heart rate were not yet affected [12].

The use of blood lactate measurement in EMS might have clinical potential: as a triage tool and as a trigger for optimisation of oxygen delivery [29-33] where the pre-hospital setting provides the earliest possible timing, which is regarded as crucial to avoid irreversible damage [34-36].

This study has several limitations. First, an important limitation is that the data were collected in 1997 and 1998. Due to practical reasons, these data have not been analysed and published until now. Although substantial time has elapsed, we believe that our data are still useful as differences between ambulance protocols of the study period (LPA version 4) in comparison with the current guidelines (LPA version 7) are minimal. Also, even if changes in pre-hospital treatment over the past few years would have affected the pre-hospital evolution of lactate, we still assume that the intrinsic association between a certain lactate course and its related impact on outcome remains unaltered. Furthermore, the impact of in-hospital care on mortality was limited because the average time to in-hospital death was only three days. Nonetheless, progress over the years in in-hospital care in the fields of emergency medicine and critical care medicine may affect the rate of in-hospital mortality.

Second, in this pilot study, we chose to include patients based on abnormal vital signs rather than including all patients for whom ambulances were dispatched. This allowed establishing associations between lactate levels, abnormalities in vital signs and outcome without needing to enroll a very large cohort of patients. However, this resulted in a relatively high mortality rate (26%), limiting the ability of the result to be generalised to other out-of-hospital settings. Also, stratified analyses of more homogeneous groups, such as trauma or medical patients, were not possible.

Last, the chosen entry criteria are compensatory mechanisms for hypoperfusion and may have confounded the potential to discover hyperlactataemia in haemodynamically stable patients. By adjusting for vital parameters in multivariable analysis and by excluding cardiac arrest patients, who are in apparent shock, we tried to correct for this.

## Conclusion

The present data show that pre-hospital blood lactate levels predicted in-hospital mortality in a population that required urgent ambulance dispatching, and that these measurements provided prognostic information over and above common vital signs. In the early pre-hospital phase, meausring lactate level was a more sensitive way of identifying a population at risk than measuring systolic blood pressure and heart rate. Its use in EMS has the potential for earlier detection of occult shock, optimisation of triage decisions and earlier start of goal-directed therapy. However, external validation in larger cohorts of consecutive patients for which ambulances are dispatched is required before widespread implementation of lactate level measurement in the out-of-hospital setting.

## Key messages

• Pre-hospital blood lactate levels were associated with in-hospital mortality.

• A blood lactate level of 3.5 mmol/L was the best cut-off value in the pre-hospital phase to discriminate survivors from non-survivors.

• The prognostic value of pre-hospital blood lactate level was superior to that of heart rate and systolic blood pressure.

• The use of blood lactate measurement in EMS might have potential for triage decisions, earlier detection of occult shock and earlier start of goal-directed therapy.

## Abbreviations

AUROC: area under the ROC curve; CI: confidence interval; ED: emergency department; EMS: Emergency Medical Services; GCS: Glasgow Coma Scale; ICU: intensive care unit; LPA: Landelijk Protocol Ambulancezorg (Dutch ambulance protocols); NPV: negative predictive value; PH: proportional hazards; PPV: positive predictive value; ROC: receiver operating characteristic; SD: standard deviation; SpO_2_: peripheral oxygen saturation obtained by pulseoxymeter; T1: on arrival of the ambulance at the scene; T2: just before or on arrival at the emergency department.

## Competing interests

The authors have no conflicts of interest. The study was supported by Roche Diagnostics (Mannheim, Germany), which provided the Accutrend hand-held lactate analysers.

## Authors' contributions

TJ analysed and interpreted data, and drafted the manuscript. JvB interpreted data and helped to draft the manuscript. PM performed the statistical analyses. JR and SS conceived the study. JB conceived and co-ordinated the study, and revised the manuscript. TJ, JvB and JB took responsibility for the paper as a whole.
